# Supplementary material for: Association of immediate versus delayed extubation of patients admitted to intensive care units postoperatively and outcomes: A retrospective study
Source: PLoS One. 2023 Jan 23;18(1):e0280820. doi: 10.1371/journal.pone.0280820 (PMC9870150; doi:10.1371/journal.pone.0280820)
Supplement: S1 File — (PDF) [file pone.0280820.s002.pdf]

SUPPLEMENTARY MATERIALS

Association of immediate versus delayed extubation of patients admitted to intensive care units postoperatively and outcomes: a retrospective study

Paul Zajic, MD PhD
Division of General Anaesthesiology, Emergency- and Intensive Care Medicine, Medical University of Graz, Graz, Austria

Michael Eichinger, MD
Division of General Anaesthesiology, Emergency- and Intensive Care Medicine, Medical University of Graz, Graz, Austria

Michael Eichlseder, MD
Division of General Anaesthesiology, Emergency- and Intensive Care Medicine, Medical University of Graz, Graz, Austria

Barbara Hallmann, MD
Division of General Anaesthesiology, Emergency- and Intensive Care Medicine, Medical University of Graz, Graz, Austria

Gabriel Honnef, MD
Division of General Anaesthesiology, Emergency- and Intensive Care Medicine, Medical University of Graz, Graz, Austria

Tobias Fellinger, BSc
Center for Medical Statistics, Informatics and Intelligent Systems, Medical University of Vienna, Vienna, Austria

Barbara Metnitz, PhD
Austrian Center for Documentation and Quality Assurance in Intensive Care, Vienna, Austria

Martin Posch, PhD
Center for Medical Statistics, Informatics and Intelligent Systems, Medical University of Vienna, Vienna, Austria

Martin Rief, MD
Division of General Anaesthesiology, Emergency- and Intensive Care Medicine, Medical University of Graz, Graz, Austria

Philipp G.H. Metnitz, MD PhD
Division of General Anaesthesiology, Emergency- and Intensive Care Medicine, Medical University of Graz, Graz, Austria

Corresponding Author

Paul Zajic, MD PhD
Division of General Anaesthesiology, Emergency- and Intensive Care Medicine, Medical University of Graz
Auenbruggerplatz 5, A-8036 Graz, Austria
paul.zajic@medunigraz.at
+43 316 385 14909

Table S 1 Baseline patient characteristics, characteristics of surgery, and outcomes in the *curtailed population*. ICU = intensive care unit, IQR = inter-quartile range, SAPS 3 = Simplified Acute Physiology Score 3, SAS = Riker Sedation and Agitation Scale

		Extubation at	
	**Total**	**Day 1 (day of admission)**	**Day 2 (1st postoperative day)**
n of patients	52562	36392	16171
Age [years] (median, IQR)	67 (56-76)	67 (55-75)	69 (57-76)
Male sex (n, %)	30651 (58%)	20895 (57%)	9756 (60%)
Type of surgery (n, %)			
Abdominal / transplant	13670 (26%)	9741 (27%)	3929 (24%)
Cardiothoracic	18615 (36%)	12525 (35%)	6090 (38%)
Neuro	8569 (16%)	6998 (19%)	1571 (10%)
Trauma / orthopedic	3197 (6%)	1927 (5%)	1270 (8%)
Other	8511 (16%)	5200 (14%)	3311 (20%)
Urgency of surgery (n, %)			
Elective surgery	38294 (73%)	28558 (79%)	9736 (60%)
Emergency surgery	12836 (24%)	6871 (19%)	5965 (37%)
Unspecified	1432 (3%)	962 (2%)	470 (3%)
SAPS 3 score (median, IQR)	43 (36-51)	42 (35-50)	46 (39-55)
Time of ICU admission (n, %)			
00:00 – 07:59	4230 (8%)	3341 (9%)	889 (5%)
08:00 – 15:59	37599 (72%)	28434 (78%)	9165 (57%)
16:00 – 23:59	10733 (20%)	4616 (13%)	6117 (38%)
ICU length of stay [days] (median, IQR)	3 (2-4)	2 (2-4)	3 (2-5)
Hospital length of stay [days] (median, IQR)	15 (10-22)	14 (10-21)	16 (11-26)
Re-intubation during ICU stay (n, %)	1968 (4%)	1151 (3%)	817 (5%)
In-hospital mortality (n, %)	2109 (4%)	1151 (3%)	958 (6%)
Agitation or over-sedation during ICU stay after extubation			
SAS>5 or SAS<3	1077 (2%	205 (1%)	613 (4%)
SAS>4 or SAS<4	4470 (9%)	1854 (5%)	21616 (16%)

Table S 2 Baseline patient characteristics, characteristics of surgery, and outcomes in the *target trial population*. ICU = intensive care unit, IQR = inter-quartile range, SAPS 3 = Simplified Acute Physiology Score 3, SAS = Riker Sedation and Agitation Scale

		Extubation at	
	**Total**	**Day 1 (day of admission)**	**Day 2 (1st postoperative day or after)**
n of patients	33990	24421	9569
Age [years] (median, IQR)	67 (56-75)	67 (55-75)	69 (59-76)
Male sex (n, %)	19550 (58%)	13780 (56%)	5770 (60%)
Type of surgery (n, %)			
Abdominal / transplant	8340 (25%)	6448 (26%)	1892 (20%)
Cardiothoracic	15882 (47%)	10247 (42%)	5635 (59%)
Neuro	7097 (21%)	5904 (24%)	1193 (12%)
Trauma / orthopedic	1516 (4%)	967 (4%)	549 (6%)
Other	1155 (3%)	855 (4%)	300 (3%)
Urgency of surgery (n, %)			
Elective surgery	31524 (93%)	22911 (94%)	8613 (90%)
Emergency surgery	1511 (4%)	864 (3%)	647 (7%)
Unspecified	955 (3%)	646 (3%)	309 (3%)
SAPS 3 score (median, IQR)	40 (34-47)	40 (34-46)	42 (36-48)
Time of ICU admission (n, %)			
00:00 – 07:59	1935 (6%)	1526 (6%)	409 (4%)
08:00 – 15:59	27958 (82%)	21022 (86%)	6936 (73%)
16:00 – 23:59	4097 (12%)	1873 (8%)	2224 (23%)
Extubation at			
Day of ICU admission	24421 (72%)	24421 (100%)	0 (0%)
1st postoperative day	6606 (19%)	0 (0%)	6606 (69%)
After 1st postoperative day	2963 (9%)	0 (0%)	2963 (31%)
ICU length of stay [days] (median, IQR)	2 (2-4)	2 (2-3)	4 (2-7)
Hospital length of stay [days] (median, IQR)	14 (10-21)	14 (10-20)	17 (12-26)
Re-intubation during ICU stay (n, %)	900 (3%)	510 (2%)	390 (4%)
In-hospital mortality (n, %)	1045 (3%)	426 (2%)	619 (7%)
Agitation or over-sedation during ICU stay after extubation			
SAS>5 or SAS<3	1270 (4%)	232 (1%)	1038 (11%)
SAS>4 or SAS<4	3706 (11%)	932 (4%)	2774 (29%)

Table S 3 Multivariate logistic regression analysis performed in the *main population* for ***re-intubation*** as the dependent variable and variables of interest and adjustment as co-variates. The model includes anonymous ICU identifiers as fixed effects, these are not depicted. AUC = 0.756. CI = confidence interval, ICU = intensive care unit, OR = odds ratio, SAPS 3 = Simplified Acute Physiology Score 3

**Variable**	**OR**	**95% CI**	***p***
Extubation at			
Day 1 (day of ICU admission)	1.00		
Day 2 (1st postoperative day)	1.22	1.05-1.42	0.002
SAPS 3 score	1.03	1.02-1.03	<0.001
Type of surgery			
Abdominal / transplant	1.00		
Cardiothoracic	0.57	0.45-0.74	<0.001
Neuro	0.39	0.27-0.57	<0.001
Trauma / orthopedic	0.61	0.45-0.81	<0.001
Other	0.44	0.35-0.56	<0.001
Urgency of surgery			
Elective surgery	0.59	0.49-0.71	<0.001
Emergency surgery	1.00		
Unspecified	1.06	0.66-1.69	>0.999
Time of ICU admission			
00:00 – 07:59	1.00		
08:00 – 15:59	0.76	0.61-0.95	0.006
16:00 – 23:59	0.70	0.55-0.89	<0.001

Table S 4 Multivariate Cox cause-specific hazard regression analysis performed in the *main population* for ***ICU discharge*** as the dependent variable and variables of interest and adjustment as co-variates. Patients dying before ICU discharge were censored. The model includes anonymous ICU identifiers as fixed effects, these are not depicted. CI = confidence interval, HR = hazard ratio, ICU = intensive care unit, SAPS 3 = Simplified Acute Physiology Score 3

**Variable**	**HR**	**95% CI**	***p***
Extubation at			
Day 1 (day of ICU admission)	1.00		
Day 2 (1st postoperative day)	0.66	0.60-0.73	0.002
SAPS 3 score	0.98	0.98-0.99	<0.001
Type of surgery			
Abdominal / transplant	1.00		
Cardiothoracic	1.27	1.08-1.48	<0.001
Neuro	1.54	1.12-2.11	0.002
Trauma / orthopedic	1.12	0.99-1.26	0.108
Other	1.49	1.35-1.64	<0.001
Urgency of surgery			
Elective surgery	1.18	1.02-1.37	0.013
Emergency surgery	1.00		
Unspecified	0.99	0.79-1.24	>0.999
Time of ICU admission			
00:00 – 07:59	1.00		
08:00 – 15:59	1.05	1.00-1.11	0.071
16:00 – 23:59	1.03	0.95-1.11	0.945

Table S 5 Multivariate logistic regression analysis performed in the *main population* (*n*=49 882 due to 3 100 missing observations) for ***agitation (SAS>4) or over-sedation (SAS<4)*** during ICU stay after extubation as the dependent variable and variables of interest and adjustment as co-variates. The model includes anonymous ICU identifiers as fixed effects, these are not depicted. AUC = 0.765. CI = confidence interval, ICU = intensive care unit, OR = odds ratio, SAPS 3 = Simplified Acute Physiology Score 3, SAS = Riker Sedation and Agitation Scale

**Variable**	**OR**	**95% CI**	***p***
Extubation at			
Day 1 (day of ICU admission)	1.00		
Day 2 (1st postoperative day)	2.86	2.57-3.19	<0.001
SAPS 3 score	1.02	1.01-1.03	<0.001
Age [years]	1.01	1.01-1.02	<0.001
Serum creatinine at ICU admission [mg/dl]	1.06	1.01-1.11	<0.008
Alcoholism	1.65	1.27-2.15	<0.001
Type of surgery			
Abdominal / transplant	1.00		
Cardiothoracic	0.97	0.81-1.17	>0.999
Neuro	1.14	0.89-1.47	0.814
Trauma / orthopedic	1.31	1.06-1.63	0.003
Other			
Urgency of surgery			
Elective surgery	0.78	0.68-0.90	<0.001
Emergency surgery	1.00		
Unspecified	1.28	0.91-1.79	0.364
Time of ICU admission			
00:00 – 07:59	1.00		
08:00 – 15:59	0.83	0.69-0.99	0.036
16:00 – 23:59	0.90	0.74-1.09	0.732

Table S 6 Ordinal regression analysis performed in the *main population* (n=49 882 due to 3 100 missing observations) for ***changes from 4 on the SAS*** during ICU stay after extubation as the dependent variable and variables of interest and adjustment as co-variates. The model includes anonymous ICU identifiers as fixed effects, these are not depicted. CI = confidence interval, ICU = intensive care unit, OR = odds ratio, SAPS 3 = Simplified Acute Physiology Score 3, SAS = Riker Sedation and Agitation Scale

**Variable**	**OR**	**95% CI**	***p***
Extubation at			
Day 1 (day of ICU admission)	1.00		
Day 2 (1st postoperative day)	3.22	2.78-3.73	<0.001
SAPS 3 score	1.01	1.01-1.02	<0.001
Age [years]	1.01	1.00-1.01	0.057
Serum creatinine at ICU admission [mg/dl]	1.05	0.99-1.11	0.298
Alcoholism	1.30	0.93-1.83	0.254
Type of surgery			
Abdominal / transplant	1.00		
Cardiothoracic	0.81	0.64-1.02	0.100
Neuro	1.06	0.77-1.45	>0.999
Trauma / orthopedic	0.98	0.74-1.29	>0.999
Other	0.77	0.62-0.96	0.008
Urgency of surgery			
Elective surgery	0.79	0.66-0.95	0.003
Emergency surgery	1.00		
Unspecified	1.12	0.64-1,96	>0.999
Time of ICU admission			
00:00 – 07:59	1.00		
08:00 – 15:59	0.88	0.68-1.14	0.877
16:00 – 23:59	1.06	0.82-1.38	>0.999

Table S 7 Multivariate logistic regression analysis performed in the *curtailed population* for ***in-hospital mortality*** as the dependent variable and variables of interest and adjustment as co-variates. The model includes anonymous ICU identifiers as fixed effects, these are not depicted. AUC = 0.815. CI = confidence interval, ICU = intensive care unit, OR = odds ratio, SAPS 3 = Simplified Acute Physiology Score 3

**Variable**	**OR**	**95% CI**	***p***
Extubation at			
Day 1 (day of ICU admission)	1.00		
Day 2 (1st postoperative day)	1.23	1.06-1.43	0.001
SAPS 3 score	1.07	1.07-1.08	<0.001
Type of surgery			
Abdominal / transplant	1.00		
Cardiothoracic	0.60	0.47-0.77	<0.001
Neuro	0.35	0.24-0.50	<0.001
Trauma / orthopedic	1.10	0.86-1.41	0.958
Other	0.60	0.49-0.75	<0.001
Urgency of surgery			
Elective surgery	0.96	0.81-1.15	>0.999
Emergency surgery	1.00		
Unspecified	1.58	0.99-2.52	0.064
Time of ICU admission			
00:00 – 07:59	1.00		
08:00 – 15:59	0.97	0.76-1.24	>0.999
16:00 – 23:59	1.00	0.78-1.28	>0.999

Table S 8 Multivariate logistic regression analysis performed in the *curtailed population* for ***re-intubation*** as the dependent variable and variables of interest and adjustment as co-variates. The model includes anonymous ICU identifiers as fixed effects, these are not depicted. AUC = 0.756. CI = confidence interval, ICU = intensive care unit, OR = odds ratio, SAPS 3 = Simplified Acute Physiology Score 3

**Variable**	**OR**	**95% CI**	***p***
Extubation at			
Day 1 (day of ICU admission)	1.00		
Day 2 (1st postoperative day)	1.20	1.03-1.39	0.008
SAPS 3 score	1.03	1.02-1.04	<0.001
Type of surgery			
Abdominal / transplant	1.00		
Cardiothoracic	0.57	0.45-0.74	<0.001
Neuro	0.40	0.28-0.57	<0.001
Trauma / orthopedic	0.61	0.46-0.81	<0.001
Other	0.45	0.35-0.57	<0.001
Urgency of surgery			
Elective surgery	0.59	0.49-0.70	<0.001
Emergency surgery	1.00		
Unspecified	1.05	0.65-1.68	>0.999
Time of ICU admission			
00:00 – 07:59	1.00		
08:00 – 15:59	0.76	0.61-0.95	0.007
16:00 – 23:59	0.70	0.55-0.89	<0.001

Table S 9 Multivariate logistic regression analysis performed in the *curtailed population* (*n*=49 528 due to 3 034 missing observations) for ***agitation (SAS>5) or over-sedation (SAS<3)*** during ICU stay after extubation as the dependent variable and variables of interest and adjustment as co-variates. The model includes anonymous ICU identifiers as fixed effects, these are not depicted. AUC = 0.795. CI = confidence interval, ICU = intensive care unit, OR = odds ratio, SAPS 3 = Simplified Acute Physiology Score 3, SAS = Riker Sedation and Agitation Scale

**Variable**	**OR**	**95% CI**	***p***
Extubation at			
Day 1 (day of ICU admission)	1.00		
Day 2 (1st postoperative day)	2.12	1.72-2.62	<0.001
SAPS 3 score	1.02	1.01-1.03	<0.001
Age [years]	1.02	1.01-1.02	<0.001
Serum creatinine at ICU admission [mg/dl]	1.06	0.98-1.14	0.274
Alcoholism	1.90	1.20-3.01	0.001
Type of surgery			
Abdominal / transplant	1.00		
Cardiothoracic	1.33	0.94-1.90	0.207
Neuro	1.27	0.79-2.04	0.852
Trauma / orthopedic	1.68	1.14-2.47	0.002
Other	0.91	0.64-1.30	>0.999
Urgency of surgery			
Elective surgery	0.74	0.56-0.97	0.017
Emergency surgery	1.00		
Unspecified	1.29	0.75-2.22	0.900
Time of ICU admission			
00:00 – 07:59	1.00		
08:00 – 15:59	0.76	0.54-1.07	0.249
16:00 – 23:59	0.97	0.68-1.39	>0.999

Table S 10 Multivariate Cox cause-specific hazard regression analysis performed in the *curtailed population* for ***ICU discharge*** as the dependent variable and variables of interest and adjustment as co-variates. Patients dying before ICU discharge were censored. The model includes anonymous ICU identifiers as fixed effects, these are not depicted. CI = confidence interval, HR = hazard ratio, ICU = intensive care unit, SAPS 3 = Simplified Acute Physiology Score 3

**Variable**	**HR**	**95% CI**	***p***
Extubation at			
Day 1 (day of ICU admission)	1.00		
Day 2 (1st postoperative day)	0.67	0.61-0.74	0.002
SAPS 3 score	0.98	0.98-0.99	<0.001
Type of surgery			
Abdominal / transplant	1.00		
Cardiothoracic	1.26	1.08-1.48	<0.001
Neuro	1.53	1.13-2.08	0.001
Trauma / orthopedic	1.11	0.99-1.25	0.102
Other	1.48	1.35-1.63	<0.001
Urgency of surgery			
Elective surgery	1.19	1.03-1.38	0.007
Emergency surgery	1.00		
Unspecified	1.00	0.79-1.25	>0.999
Time of ICU admission			
00:00 – 07:59	1.00		
08:00 – 15:59	1.06	1.00-1.11	0.062
16:00 – 23:59	1.03	0.96-1.11	0.917

Table S 11 Multivariate logistic regression analysis performed in the *target trial population* for ***in-hospital mortality*** as the dependent variable and variables of interest and adjustment as co-variates. The model includes anonymous ICU identifiers as fixed effects, these are not depicted. AUC = 0.812. CI = confidence interval, ICU = intensive care unit, OR = odds ratio, SAPS 3 = Simplified Acute Physiology Score 3

**Variable**	**OR**	**95% CI**	***p***
Extubation at			
Day 1 (day of ICU admission)	1.00		
Day 2+ (1st postoperative day or later)	3.33	2.70-4.10	<0.001
SAPS 3 score	1.07	1.06-1.09	<0.001
Type of surgery			
Abdominal / transplant	1.00		
Cardiothoracic	0.47	0.33-0.67	<0.001
Neuro	0.31	0.18-0.53	<0.001
Trauma / orthopedic	1.60	1.12-2.30	0.003
Other	0.37	0.21-0.66	<0.001
Urgency of surgery			
Elective surgery	0.94	0.65-1.37	>0.999
Emergency surgery	1.00		
Unspecified	1.07	0.53-2.17	>0.999
Time of ICU admission			
00:00 – 07:59	1.00		
08:00 – 15:59	0.93	0.59-1.47	>0.999
16:00 – 23:59	0.99	0.61-1.63	>0.999

Table S 12 Multivariate logistic regression analysis performed in the *target trial population* for ***re-intubation*** as the dependent variable and variables of interest and adjustment as co-variates. The model includes anonymous ICU identifiers as fixed effects, these are not depicted. AUC = 0.753. CI = confidence interval, ICU = intensive care unit, OR = odds ratio, SAPS 3 = Simplified Acute Physiology Score 3

**Variable**	**OR**	**95% CI**	***p***
Extubation at			
Day 1 (day of ICU admission)	1.00		
Day 2+ (1st postoperative day or later)	1.74	1.40-2.18	<0.001
SAPS 3 score	1.04	1.03-1.05	<0.001
Type of surgery			
Abdominal / transplant	1.00		
Cardiothoracic	0.52	0.34-0.78	<0.001
Neuro	0.32	0.16-0.62	<0.001
Trauma / orthopedic	0.39	0.21-0.71	<0.001
Other	0.24	0.11-0.51	<0.001
Urgency of surgery			
Elective surgery	1.06	0.67-1.68	>0.999
Emergency surgery	1.00		
Unspecified	1.87	0.91-3.84	0.134
Time of ICU admission			
00:00 – 07:59	1.00		
08:00 – 15:59	0.67	0.44-1.02	0.077
16:00 – 23:59	0.84	0.52-1.35	0.969

Table S 13 Multivariate logistic regression analysis performed in the *target trial population* (*n*=32 285 due to 1 705 missing observations) for ***agitation (SAS>5) or over-sedation (SAS<3)*** during ICU stay after extubation as the dependent variable and variables of interest and adjustment as co-variates. The model includes anonymous ICU identifiers as fixed effects, these are not depicted. AUC = 0.850. CI = confidence interval, ICU = intensive care unit, OR = odds ratio, SAPS 3 = Simplified Acute Physiology Score 3, SAS = Riker Sedation and Agitation Scale

**Variable**	**OR**	**95% CI**	***p***
Extubation at			
Day 1 (day of ICU admission)	1.00		
Day 2+ (1st postoperative day or later)	10.84	8.54-13.76	<0.001
SAPS 3 score	1.02	1.01-1.04	<0.001
Age [years]	1.01	1.00-1.02	0.027
Serum creatinine at ICU admission [mg/dl]	1.11	1.00-1.23	0.074
Alcoholism	1.98	1.15-3.42	0.004
Type of surgery			
Abdominal / transplant	1.00		
Cardiothoracic	1.19	0.80-1.77	0.937
Neuro	1.09	0.59-2.01	>0.999
Trauma / orthopedic	1.18	0.69-2.04	0.997
Other	1.06	0.57-1.99	>0.999
Urgency of surgery			
Elective surgery	0.83	0.55-1.26	0.941
Emergency surgery	1.00		
Unspecified	1.26	0.69-2.32	0.978
Time of ICU admission			
00:00 – 07:59	1.00		
08:00 – 15:59	0.88	0.59-1.32	0.997
16:00 – 23:59	0.91	0.58-1.44	>0.999
