# Supplementary material for: Association of immediate versus delayed extubation of patients admitted to intensive care units postoperatively and outcomes: A retrospective study
Source: PLoS One. 2023 Jan 23;18(1):e0280820. doi: 10.1371/journal.pone.0280820 (PMC9870150; doi:10.1371/journal.pone.0280820)
Supplement: Attachment — Submitted filename: ReviewerMaterials.pdf [file pone.0280820.s003.pdf]

REVIEWER MATERIALS

Association of immediate versus delayed extubation of patients admitted to intensive care units postoperatively and outcomes: a retrospective study

Paul Zajic, MD PhD
Division of General Anaesthesiology, Emergency- and Intensive Care Medicine, Medical University of Graz, Graz, Austria

Michael Eichinger, MD
Division of General Anaesthesiology, Emergency- and Intensive Care Medicine, Medical University of Graz, Graz, Austria

Michael Eichlseder, MD
Division of General Anaesthesiology, Emergency- and Intensive Care Medicine, Medical University of Graz, Graz, Austria

Barbara Hallmann, MD
Division of General Anaesthesiology, Emergency- and Intensive Care Medicine, Medical University of Graz, Graz, Austria

Gabriel Honnef, MD
Division of General Anaesthesiology, Emergency- and Intensive Care Medicine, Medical University of Graz, Graz, Austria

Tobias Fellinger, BSc
Center for Medical Statistics, Informatics and Intelligent Systems, Medical University of Vienna, Vienna, Austria

Barbara Metnitz, PhD
Austrian Center for Documentation and Quality Assurance in Intensive Care, Vienna, Austria

Martin Posch, PhD
Center for Medical Statistics, Informatics and Intelligent Systems, Medical University of Vienna, Vienna, Austria

Martin Rief, MD
Division of General Anaesthesiology, Emergency- and Intensive Care Medicine, Medical University of Graz, Graz, Austria

Philipp G.H. Metnitz, MD PhD
Division of General Anaesthesiology, Emergency- and Intensive Care Medicine, Medical University of Graz, Graz, Austria

Corresponding Author

Paul Zajic, MD PhD
Division of General Anaesthesiology, Emergency- and Intensive Care Medicine, Medical University of Graz
Auenbruggerplatz 5, A-8036 Graz, Austria
paul.zajic@medunigraz.at
+43 316 385 14909

Table R 1 Comparison of frequencies of day 1 and day 2 extubation, respectively, throughout the years of the study period. ICU = intensive care unit

	**Extubation at**		
**Variable**	**Day 1 (day of ICU admission)**	**Day 2 (1st postoperative day)**	**Total**
Year (n, %)	4,204 (67%)	2,050 (33%)	6,254 (100%)
2012	4,855 (70%)	2,036 (30%)	6,891 (100%)
2013	4,830 (69%)	2,125 (31%)	6,955 (100%)
2014	5,289 (69%)	2,327 (31%)	7,616 (100%)
2015	4,783 (68%)	2,279 (32%)	7,062 (100%)
2016	4,817 (70%)	2,019 (30%)	6,836 (100%)
2017	4,868 (69%)	2,192 (31%)	7,060 (100%)
2018	3,165 (73%)	1,143 (27%)	4,308 (100%)
2019	4,204 (67%)	2,050 (33%)	6,254 (100%)

Univariate logistic regression analyses *not* including ICU-IDs as fixed effects

Table R 2 Univariate logistic regression analysis for ***in-hospital mortality*** as the dependent variable AUC = 0.569. CI = confidence interval, ICU = intensive care unit, OR = odds ratio

**Variable**	**OR**	**95% CI**	***p***
Extubation at			
Day 1 (day of ICU admission)	1.00		
Day 2 (1st postoperative day)	1.82	1.65-2.00	<0.001

Table R 3 Univariate logistic regression analysis for ***in-hospital mortality*** as the dependent variable AUC = 0.776. CI = confidence interval, OR = odds ratio, SAPS 3 = Simplified Acute Physiology Score 3

**Variable**	**OR**	**95% CI**	***p***
SAPS 3 score	1.08	1.08-1.09	<0.001

Table R 4 Univariate logistic regression analysis for ***in-hospital mortality*** as the dependent variable AUC = 0.579. CI = confidence interval, ICU = intensive care unit, OR = odds ratio

**Variable**	**OR**	**95% CI**	***p***
Time of ICU admission			
00:00 – 07:59	1.00		
08:00 – 15:59	0.61	0.52-0.71	<0.001
16:00 – 23:59	1.27	1.06-1.50	0.005

Table R 5 Univariate logistic regression analysis for ***in-hospital mortality*** as the dependent variable AUC = 0.647. CI = confidence interval, OR = odds ratio

**Variable**	**OR**	**95% CI**	***p***
Urgency of surgery			
Elective surgery	0.28	0.21-0.31	<0.001
Emergency surgery	1.00		
Unspecified	0.67	0.51-0.88	0.002

Table R 6 Univariate logistic regression analysis for ***in-hospital mortality*** as the dependent variable

AUC = 0.656. CI = confidence interval, OR = odds ratio

**Variable**	**OR**	**95% CI**	***p***
Type of surgery			
Abdominal / transplant	1.00		
Cardiothoracic	0.28	0.24-0.33	<0.001
Neuro	0.24	0.19-0.29	<0.001
Trauma / orthopedic	0.91	0.75-1.10	0.633
Other	0.58	0.50-0.68	<0.001

Univariate logistic regression analyses including ICU-IDs as fixed effects

Table R 7 Univariate logistic regression analysis for ***in-hospital mortality*** as the dependent variable AUC = 0.722. CI = confidence interval, ICU = intensive care unit, OR = odds ratio

Variable	OR	95% CI	*p*
Extubation at			
Day 1 (day of ICU admission)	1.00		
Day 2 (1st postoperative day)	1.59	1.43-1.77	<0.001

Table R 8 Univariate logistic regression analysis for ***in-hospital mortality*** as the dependent variable AUC = 0.810. CI = confidence interval, OR = odds ratio, SAPS 3 = Simplified Acute Physiology Score 3

Variable	OR	95% CI	*p*
SAPS 3 score	1.08	1.07-1.08	<0.001

Table R 9 Univariate logistic regression analysis for ***in-hospital mortality*** as the dependent variable AUC = 0.721. CI = confidence interval, ICU = intensive care unit, OR = odds ratio

Variable	OR	95% CI	*p*
Time of ICU admission			
00:00 – 07:59	1.00		
08:00 – 15:59	0.66	0.55-0.79	<0.001
16:00 – 23:59	1.02	0.85-1.24	0.984

Table R 10 Univariate logistic regression analysis for ***in-hospital mortality*** as the dependent variable AUC = 0.742. CI = confidence interval, OR = odds ratio

Variable	OR	95% CI	*p*
Urgency of surgery			
Elective surgery	0.38	0.34-0.43	<0.001
Emergency surgery	1.00		
Unspecified	0.68	0.47-0.97	0.029

Table R 11 Univariate logistic regression analysis for ***in-hospital mortality*** as the dependent variable

AUC = 0.656. CI = confidence interval, OR = odds ratio

**Variable**	**OR**	**95% CI**	***p***
Type of surgery			
Abdominal / transplant	1.00		
Cardiothoracic			<0.001
Neuro			<0.001
Trauma / orthopedic			0.633
Other			<0.001
